# Supplementary material for: Cardiovascular Disease-Associated MicroRNAs as Novel Biomarkers of First-Trimester Screening for Gestational Diabetes Mellitus in the Absence of Other Pregnancy-Related Complications
Source: Int J Mol Sci. 2022 Sep 13;23(18):10635. doi: 10.3390/ijms231810635 (PMC9501303; doi:10.3390/ijms231810635)
Supplement: Supplementary file 1 [file ijms-23-10635-s001.zip › Supplementary Figure S1.pdf]

Supplementary Figure S1.

A

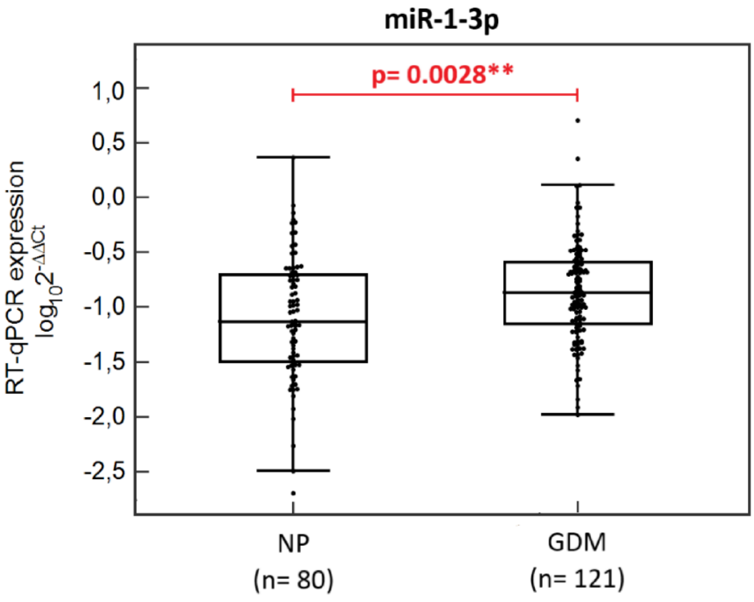

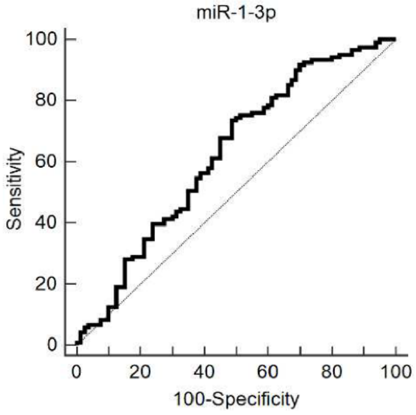

Area under the ROC curve (AUC)	0.625
Standard Error	0.0415
95% Confidence interval	0.554 to 0.692
Significance level P (Area=0.5)	0.0027

Estimated sensitivity at fixed specificity			
Specificity	Sensitivity	95% CI	Criterion
90.00	12.40	4.13 to 36.36	>0.377841

Criterion	Sensitivity	95% CI	Specificity	95% CI	+LR	95% CI	-LR	95% CI
>0.075962	73.55	64.8 - 81.2	51.25	39.8 - 62.6	1.51	1.2 - 1.9	0.52	0.4 - 0.7

B

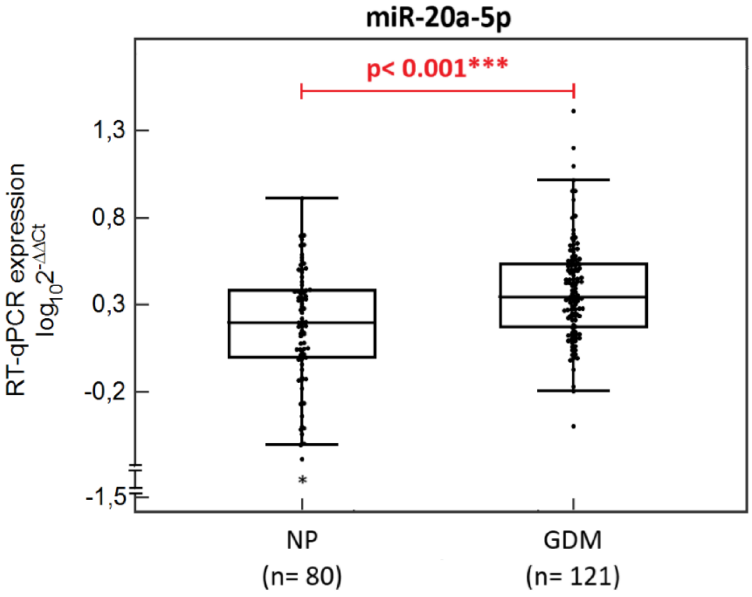

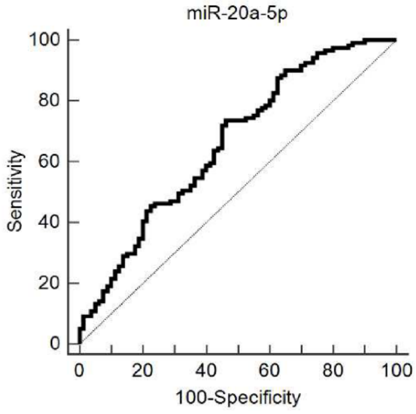

Area under the ROC curve (AUC)	0.661
Standard Error	0.0397
95% Confidence interval	0.591 to 0.726
Significance level P (Area=0.5)	0.0001

Estimated sensitivity at fixed specificity			
Specificity	Sensitivity	95% CI	Criterion
90.00	21.49	9.92 to 37.19	>3.587078

Criterion	Sensitivity	95% CI	Specificity	95% CI	+LR	95% CI	-LR	95% CI
>1.569194	73.55	64.8 - 81.2	50.00	38.6 - 61.4	1.47	1.2 - 1.9	0.53	0.4 - 0.8

C

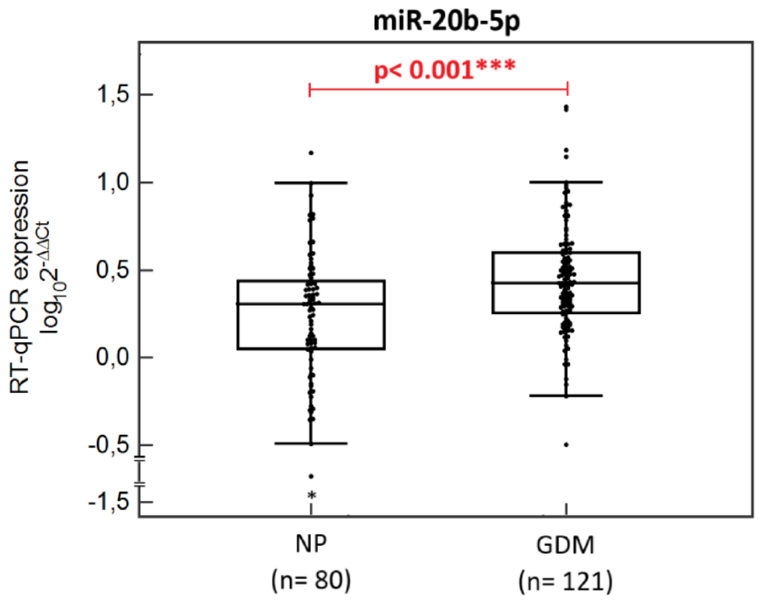

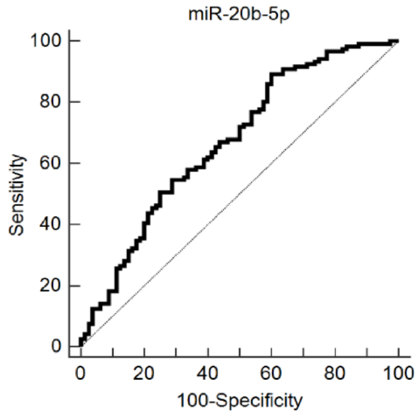

Area under the ROC curve (AUC)	0.670
Standard Error	0.0396
95% Confidence interval	0.600 to 0.734
Significance level P (Area=0.5)	<0.0001

Estimated sensitivity at fixed specificity			
Specificity	Sensitivity	95% CI	Criterion
90.00	18.18	7.44 to 33.88	>4.517659

Criterion	Sensitivity	95% CI	Specificity	95% CI	+LR	95% CI	-LR	95% CI
>1.385992	89.26	82.3 - 94.2	40.00	29.2 - 51.6	1.49	1.2 - 1.8	0.27	0.2 - 0.5

D

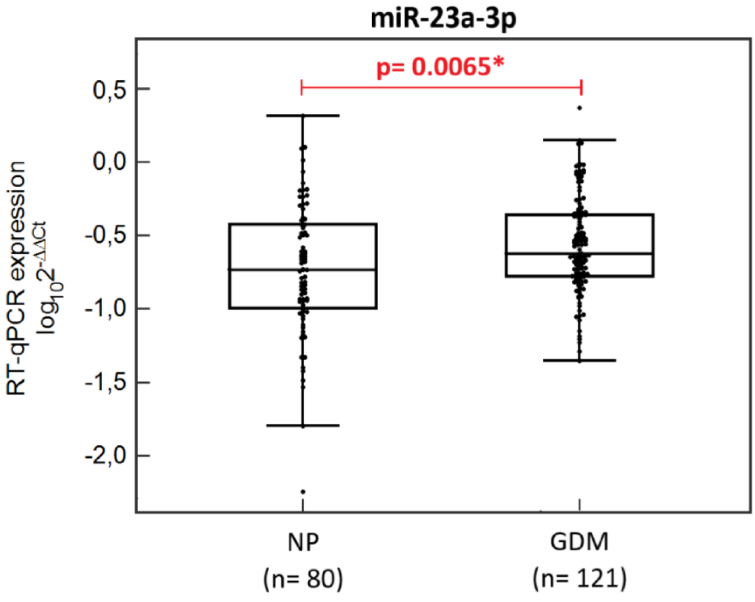

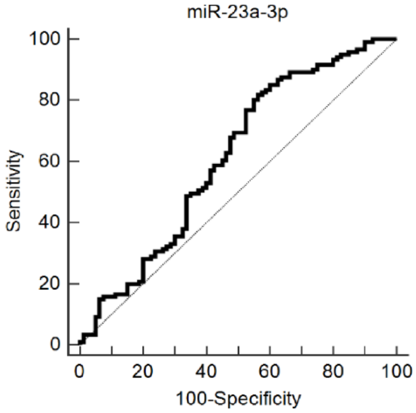

Area under the ROC curve (AUC)	0.613
Standard Error	0.0422
95% Confidence interval	0.542 to 0.681
Significance level P (Area=0.5)	0.0072

Estimated sensitivity at fixed specificity			
Specificity	Sensitivity	95% CI	Criterion
90.00	15.70	5.79 to 24.79	>0.642408

Criterion	Sensitivity	95% CI	Specificity	95% CI	+LR	95% CI	-LR	95% CI
>0.150403	81.82	73.8 - 88.2	43.75	32.7 - 55.3	1.45	1.2 - 1.8	0.42	0.3 - 0.7

E

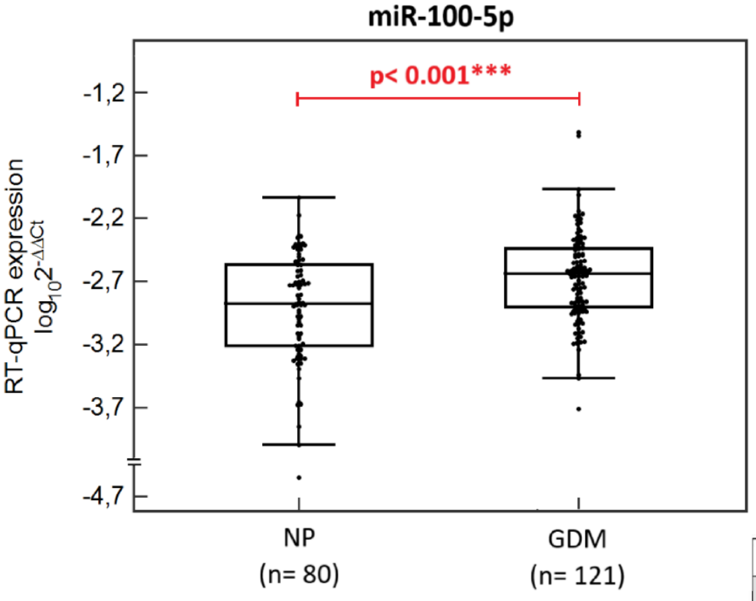

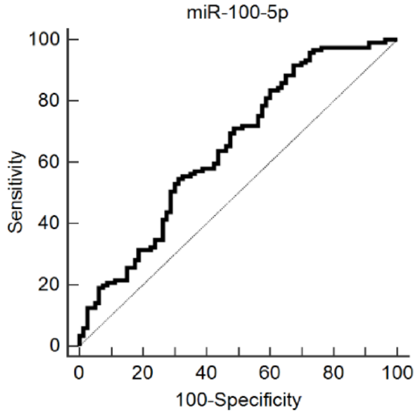

Area under the ROC curve (AUC)	0.649
Standard Error	0.0404
95% Confidence interval	0.579 to 0.715
Significance level P (Area=0.5)	0.0002

Estimated sensitivity at fixed specificity			
Specificity	Sensitivity	95% CI	Criterion
90.00	20.66	11.57 to 32.23	>0.003876

Criterion	Sensitivity	95% CI	Specificity	95% CI	+LR	95% CI	-LR	95% CI
>0.00077	91.74	85.3 - 96.0	32.50	22.4 - 43.9	1.36	1.2 - 1.6	0.25	0.1 - 0.5

F

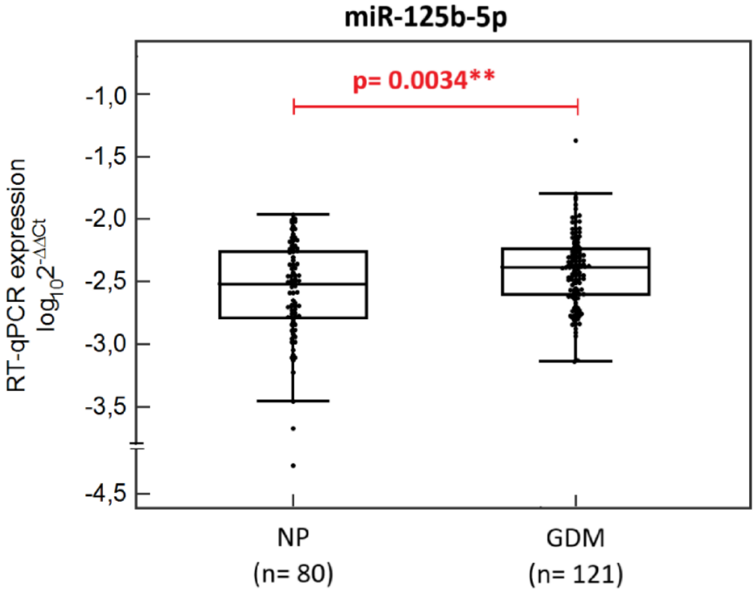

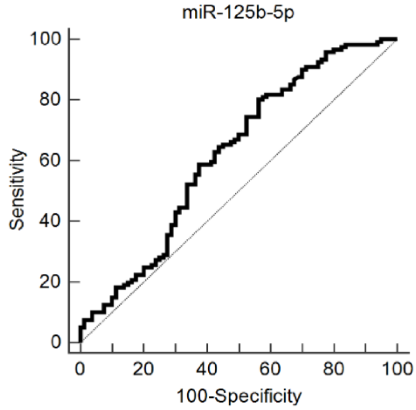

Area under the ROC curve (AUC)	0.622
Standard Error	0.0418
95% Confidence interval	0.551 to 0.689
Significance level P (Area=0.5)	0.0035

Estimated sensitivity at fixed specificity			
Specificity	Sensitivity	95% CI	Criterion
90.00	14.88	6.61 to 25.62	>0.007436

Criterion	Sensitivity	95% CI	Specificity	95% CI	+LR	95% CI	-LR	95% CI
>0.002223	80.17	71.9 - 86.9	43.75	32.7 - 55.3	1.43	1.2 - 1.8	0.45	0.3 - 0.7

G

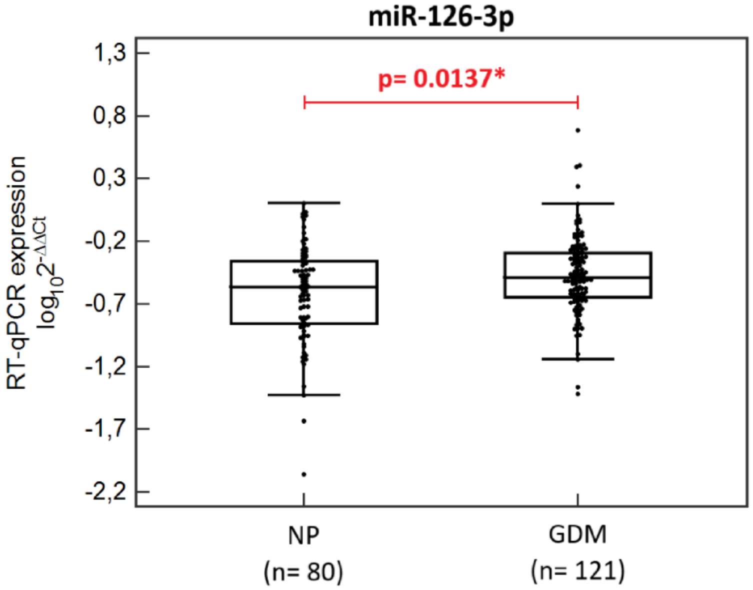

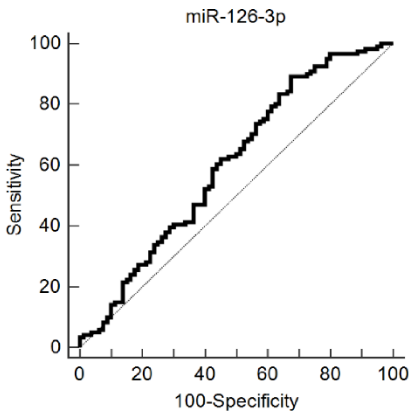

Area under the ROC curve (AUC)	0.603
Standard Error	0.0420
95% Confidence interval	0.532 to 0.671
Significance level P (Area=0.5)	0.0144

Estimated sensitivity at fixed specificity			
Specificity	Sensitivity	95% CI	Criterion
90.00	14.05	4.13 to 33.06	>0.659038

Criterion	Sensitivity	95% CI	Specificity	95% CI	+LR	95% CI	-LR	95% CI
>0.157194	89.26	82.3 - 94.2	32.50	22.4 - 43.9	1.32	1.1 - 1.6	0.33	0.2 - 0.6

H

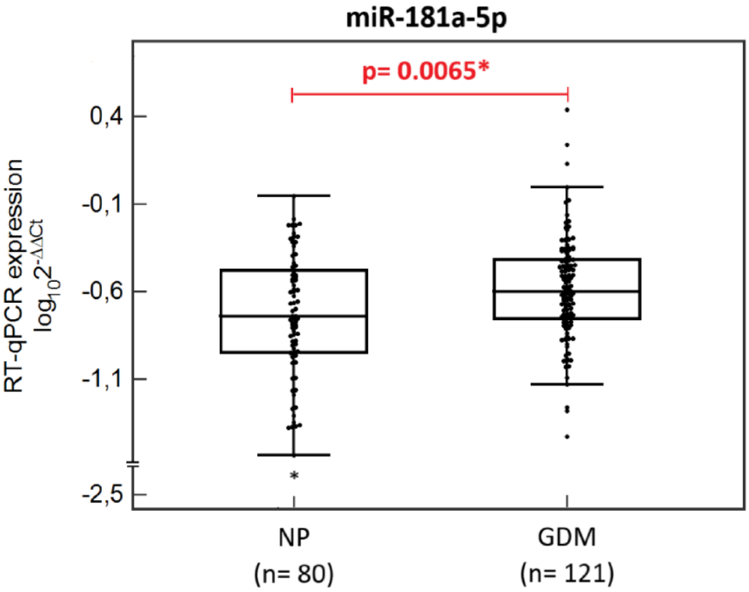

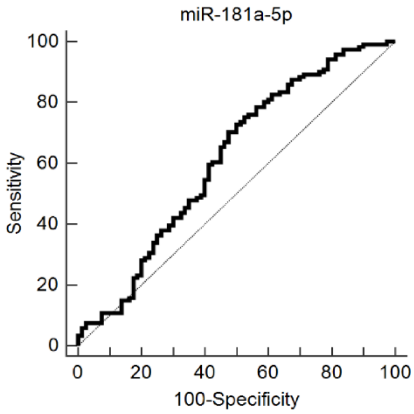

Area under the ROC curve (AUC)	0.613
Standard Error	0.0421
95% Confidence interval	0.542 to 0.681
Significance level P (Area=0.5)	0.0071

Estimated sensitivity at fixed specificity			
Specificity	Sensitivity	95% CI	Criterion
90.00	10.74	4.96 to 23.97	>0.521629

Criterion	Sensitivity	95% CI	Specificity	95% CI	+LR	95% CI	-LR	95% CI
>0.183625	70.25	61.3 - 78.2	51.25	39.8 - 62.6	1.44	1.1 - 1.9	0.58	0.4 - 0.8

I

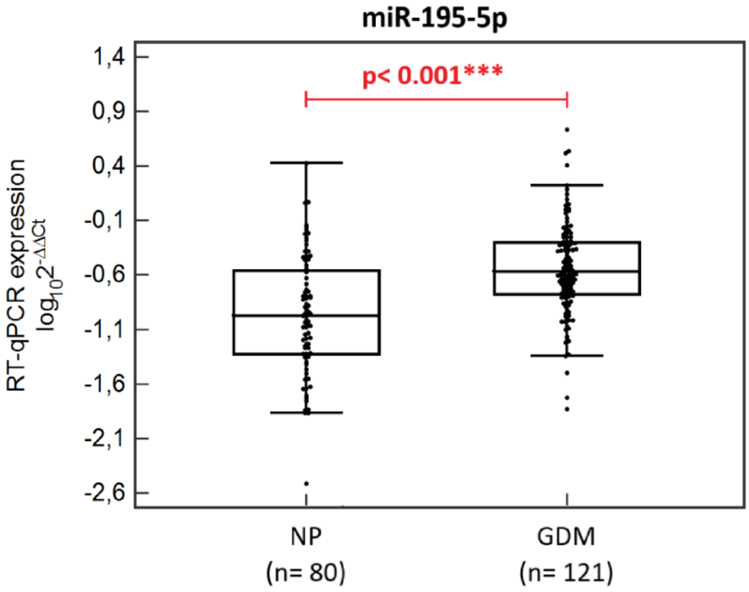

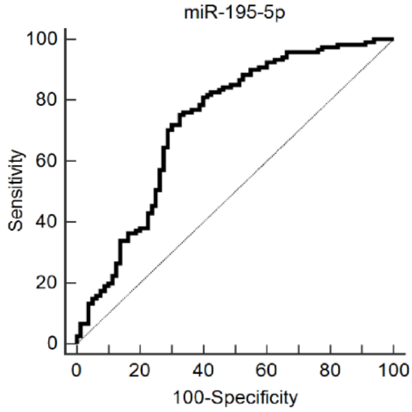

Area under the ROC curve (AUC)	0.729
Standard Error	0.0383
95% Confidence interval	0.662 to 0.789
Significance level P (Area=0.5)	<0.0001

Estimated sensitivity at fixed specificity			
Specificity	Sensitivity	95% CI	Criterion
90.00	19.83	9.41 to 38.02	>0.553901

Criterion	Sensitivity	95% CI	Specificity	95% CI	+LR	95% CI	-LR	95% CI
>0.161246	75.21	66.5 - 82.6	67.50	56.1 - 77.6	2.31	1.7 - 3.2	0.37	0.3 - 0.5

J

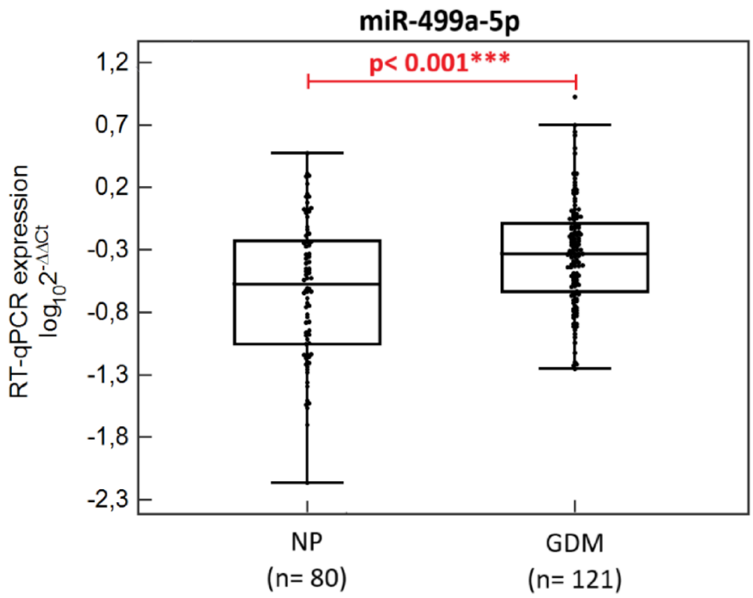

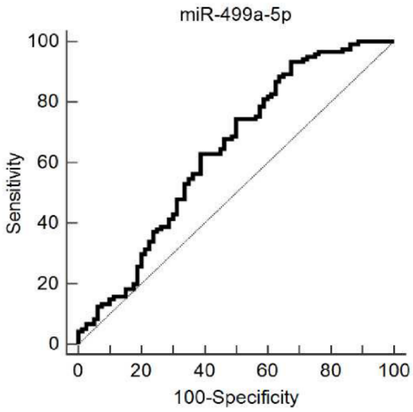

Area under the ROC curve (AUC)	0.638
Standard Error	0.0415
95% Confidence interval	0.567 to 0.704
Significance level P (Area=0.5)	0.0009

Estimated sensitivity at fixed specificity			
Specificity	Sensitivity	95% CI	Criterion
90.00	14.88	7.44 to 30.58	>1.191424

Criterion	Sensitivity	95% CI	Specificity	95% CI	+LR	95% CI	-LR	95% CI
>0.112736	93.39	87.4 - 97.1	32.50	22.4 - 43.9	1.38	1.2 - 1.6	0.20	0.10 - 0.4

K

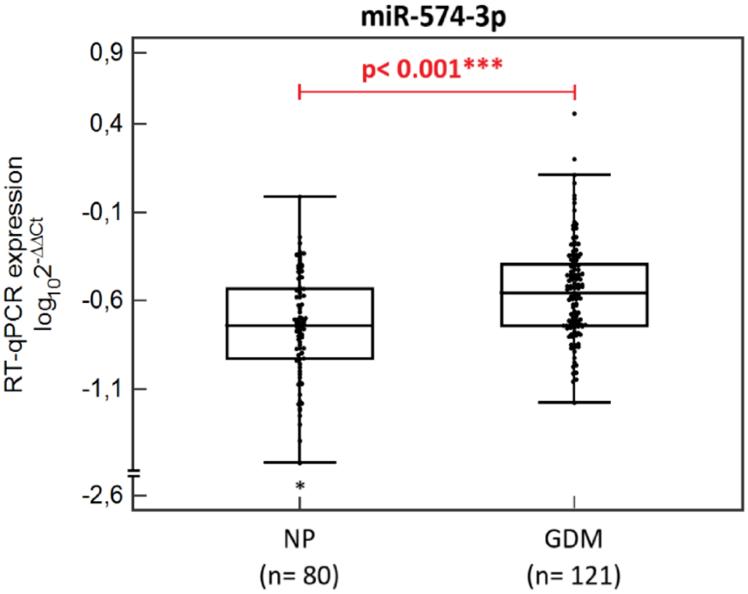

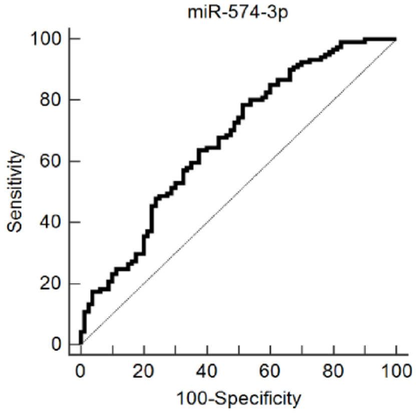

Area under the ROC curve (AUC)	0.673
Standard Error	0.0393
95% Confidence interval	0.603 to 0.737
Significance level P (Area=0.5)	<0.0001

Estimated sensitivity at fixed specificity			
Specificity	Sensitivity	95% CI	Criterion
90.00	23.14	14.05 to 38.02	>0.42623

Criterion	Sensitivity	95% CI	Specificity	95% CI	+LR	95% CI	-LR	95% CI
>0.175418	78.51	70.1 - 85.5	48.75	37.4 - 60.2	1.53	1.2 - 1.9	0.44	0.3 - 0.7

Supplementary Figure S1. Gene expression of miR-1-3p, miR-20a-5p, miR-20b-5p, miR-23a-3p, miR-100-5p, miR-125b-5p, miR-126-3p, miR-181a-5p, miR-195-5p, miR-499a-5p, and miR-574-3p in peripheral blood leukocytes in early stages of gestation (within 10 to 13 weeks) differentiates significantly (after the Benjamini-Hochberg correction) between pregnancies destinated to develop GDM and term pregnancies with normal course of gestation.
