# Supplementary material for: Cardiovascular Disease-Associated MicroRNAs as Novel Biomarkers of First-Trimester Screening for Gestational Diabetes Mellitus in the Absence of Other Pregnancy-Related Complications
Source: Int J Mol Sci. 2022 Sep 13;23(18):10635. doi: 10.3390/ijms231810635 (PMC9501303; doi:10.3390/ijms231810635)
Supplement: Supplementary file 1 [file ijms-23-10635-s001.zip › Supplementary Figure S2.pdf]

Supplementary Figure S2.

A

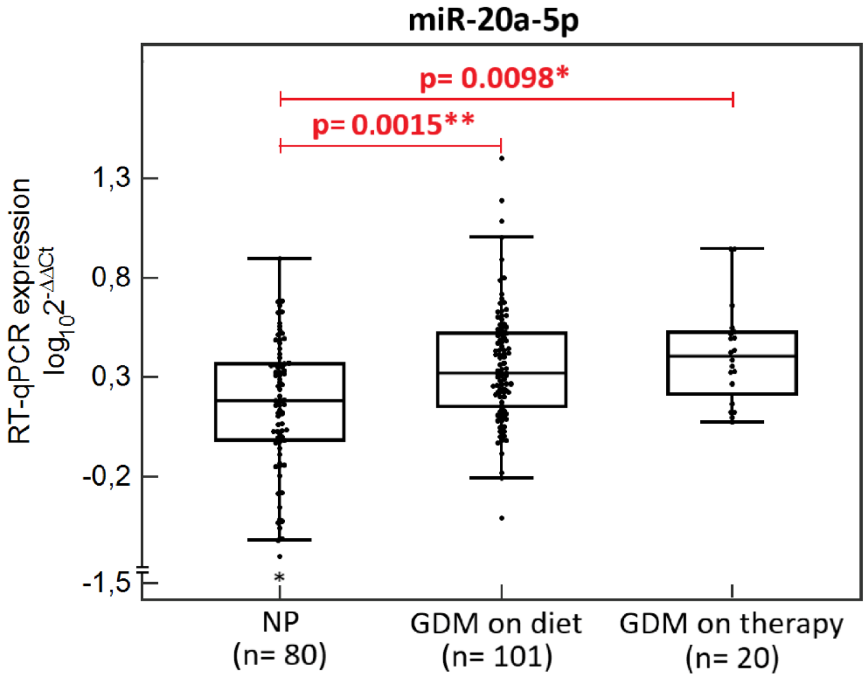

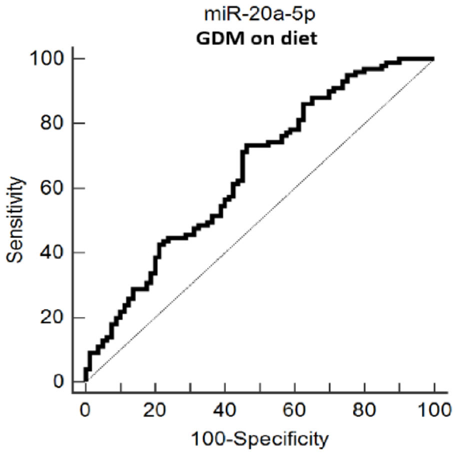

Area under the ROC curve (AUC)	0.651
Standard Error	0.0413
95% Confidence interval	0.577 to 0.720
Significance level P (Area=0.5)	0.0002

Estimated sensitivity at fixed specificity			
Specificity	Sensitivity	95% CI	Criterion
90.00	21.78	9.90 to 35.66	>3.587078

Criterion	Sensitivity	95% CI	Specificity	95% CI	+LR	95% CI	-LR	95% CI
>1.596064	73.27	63.5 - 81.6	53.75	42.2 - 65.0	1.58	1.2 - 2.1	0.50	0.3 - 0.7

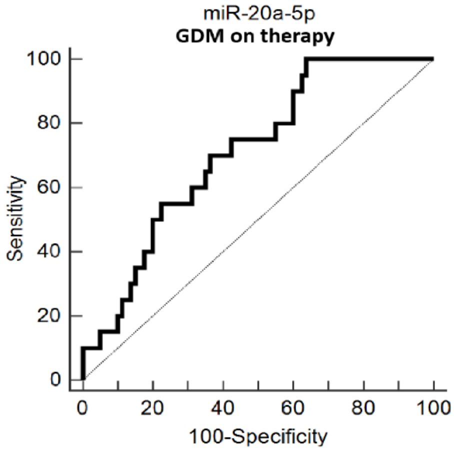

Area under the ROC curve (AUC)	0.709
Standard Error	0.0587
95% Confidence interval	0.610 to 0.796
Significance level P (Area=0.5)	0.0004

Estimated sensitivity at fixed specificity			
Specificity	Sensitivity	95% CI	Criterion
90.00	20.00	0.00 to 45.00	>3.587078

Criterion	Sensitivity	95% CI	Specificity	95% CI	+LR	95% CI	-LR	95% CI
>1.201797	100.00	83.2 - 100.0	36.25	25.8 - 47.8	1.57	1.3 - 1.9	0.00	

B

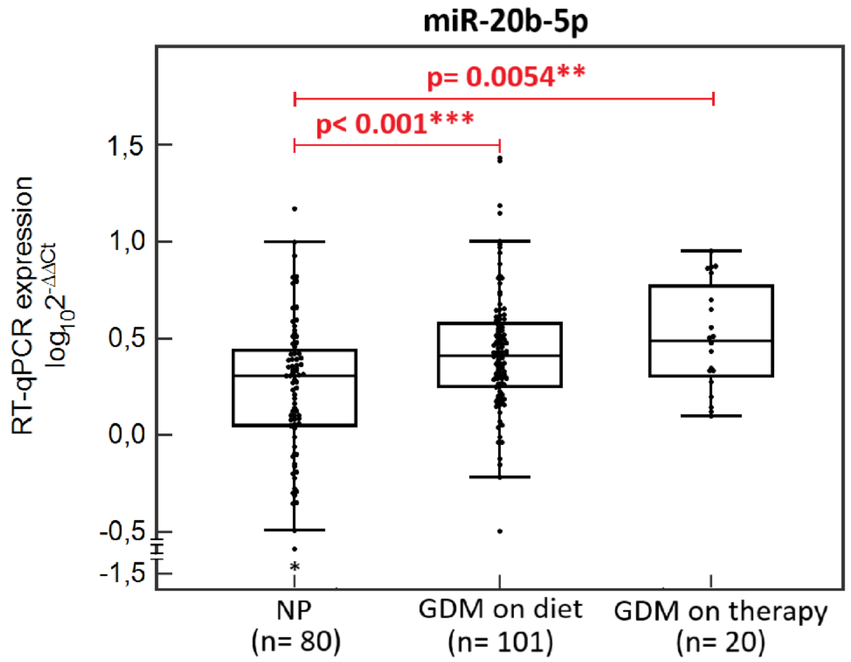

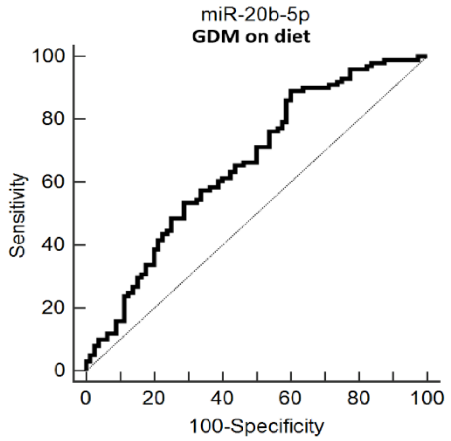

Area under the ROC curve (AUC)	0.659
Standard Error	0.0412
95% Confidence interval	0.585 to 0.728
Significance level P (Area=0.5)	0.0001

Estimated sensitivity at fixed specificity			
Specificity	Sensitivity	95% CI	Criterion
90.00	15.84	5.94 to 33.66	>4.517659

Criterion	Sensitivity	95% CI	Specificity	95% CI	+LR	95% CI	-LR	95% CI
>1.385992	89.11	81.3 - 94.4	40.00	29.2 - 51.6	1.49	1.2 - 1.8	0.27	0.1 - 0.5

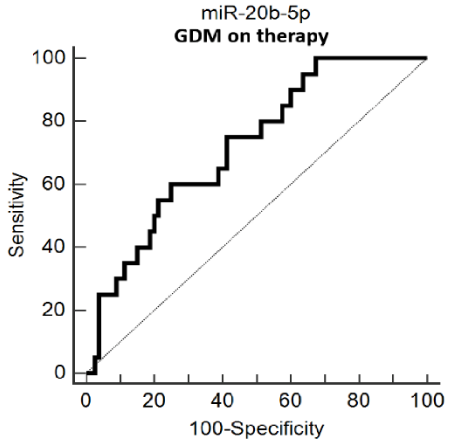

Area under the ROC curve (AUC)	0.721
Standard Error	0.0596
95% Confidence interval	0.622 to 0.806
Significance level P (Area=0.5)	0.0002

Estimated sensitivity at fixed specificity			
Specificity	Sensitivity	95% CI	Criterion
90.00	30.00	10.00 to 55.00	>4.517659

Criterion	Sensitivity	95% CI	Specificity	95% CI	+LR	95% CI	-LR	95% CI
>2.64699	60.00	36.1 - 80.9	75.00	64.1 - 84.0	2.40	1.4 - 4.0	0.53	0.3 - 0.9

C

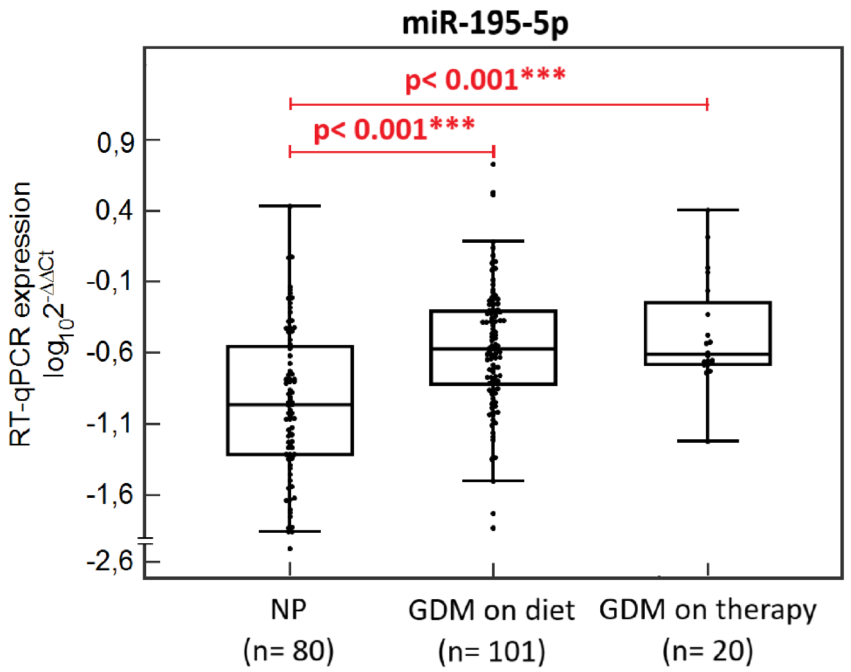

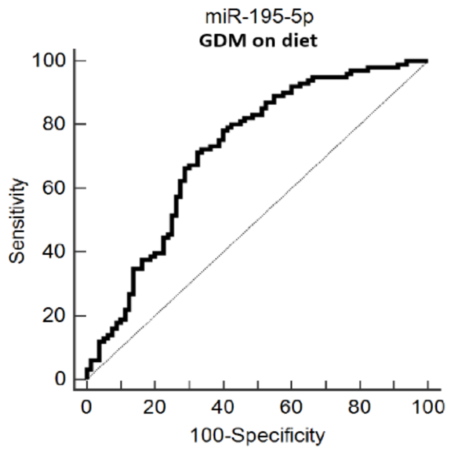

Area under the ROC curve (AUC)	0.720
Standard Error	0.0394
95% Confidence interval	0.648 to 0.784
Significance level P (Area=0.5)	<0.0001

Estimated sensitivity at fixed specificity			
Specificity	Sensitivity	95% CI	Criterion
90.00	18.81	7.92 to 39.60	>0.553901

Criterion	Sensitivity	95% CI	Specificity	95% CI	+LR	95% CI	-LR	95% CI
>0.161246	71.29	61.4 - 79.9	67.50	56.1 - 77.6	2.19	1.6 - 3.1	0.43	0.3 - 0.6

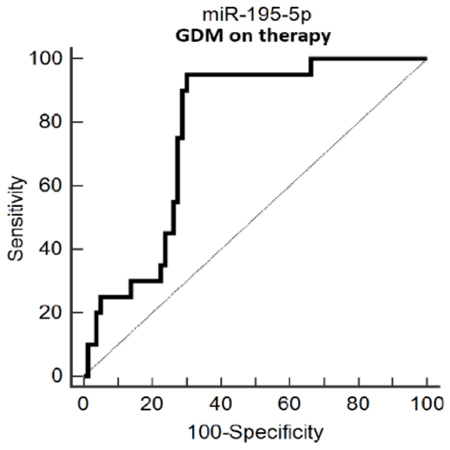

Area under the ROC curve (AUC)	0.778
Standard Error	0.0488
95% Confidence interval	0.684 to 0.855
Significance level P (Area=0.5)	<0.0001

Estimated sensitivity at fixed specificity			
Specificity	Sensitivity	95% CI	Criterion
90.00	25.00	10.00 to 45.00	>0.553901

Criterion	Sensitivity	95% CI	Specificity	95% CI	+LR	95% CI	-LR	95% CI
>0.176527	95.00	75.1 - 99.9	70.00	58.7 - 79.7	3.17	2.2 - 4.5	0.071	0.01 - 0.5

D

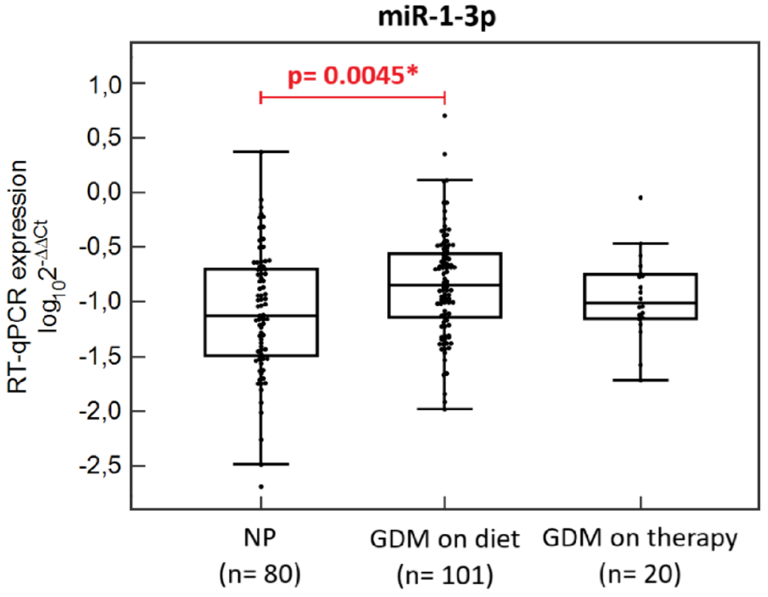

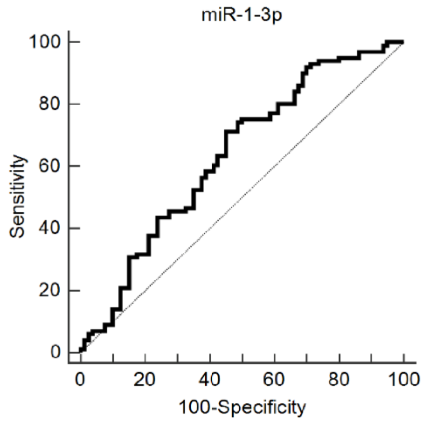

Area under the ROC curve (AUC)	0.635
Standard Error	0.0422
95% Confidence interval	0.560 to 0.705
Significance level P (Area=0.5)	0.0014

Estimated sensitivity at fixed specificity			
Specificity	Sensitivity	95% CI	Criterion
90.00	13.86	4.95 to 36.63	>0.377841

Criterion	Sensitivity	95% CI	Specificity	95% CI	+LR	95% CI	-LR	95% CI
>0.094818	71.29	61.4 - 79.9	55.00	43.5 - 66.2	1.58	1.2 - 2.1	0.52	0.4 - 0.8

E

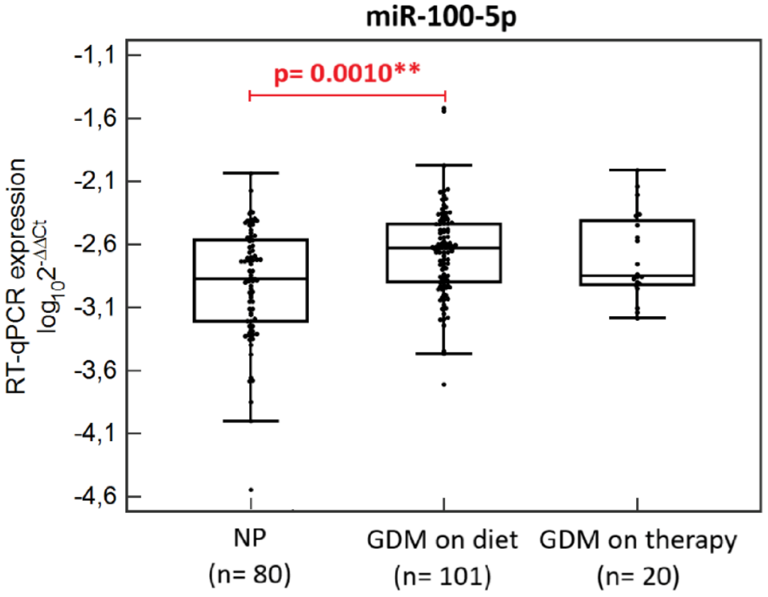

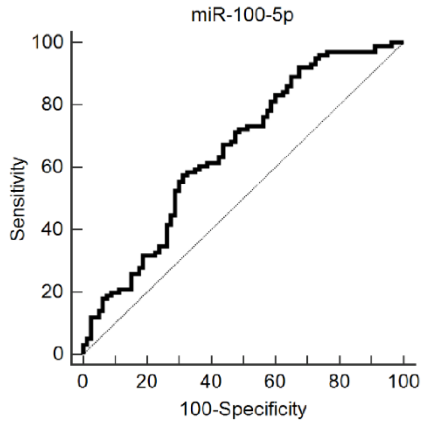

Area under the ROC curve (AUC)	0.654
Standard Error	0.0415
95% Confidence interval	0.580 to 0.723
Significance level P (Area=0.5)	0.0002

Estimated sensitivity at fixed specificity			
Specificity	Sensitivity	95% CI	Criterion
90.00	19.80	10.89 to 30.69	>0.003876

Criterion	Sensitivity	95% CI	Specificity	95% CI	+LR	95% CI	-LR	95% CI
>0.00203	57.43	47.2 - 67.2	68.75	57.4 - 78.7	1.84	1.3 - 2.6	0.62	0.5 - 0.8

F

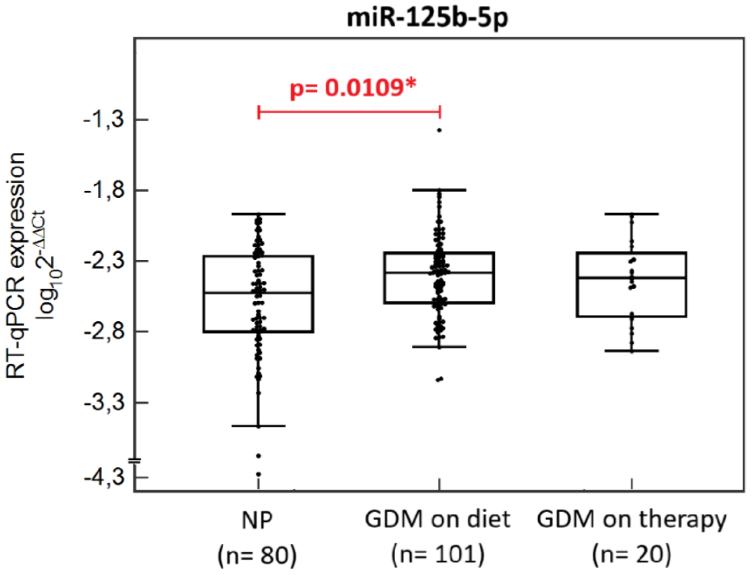

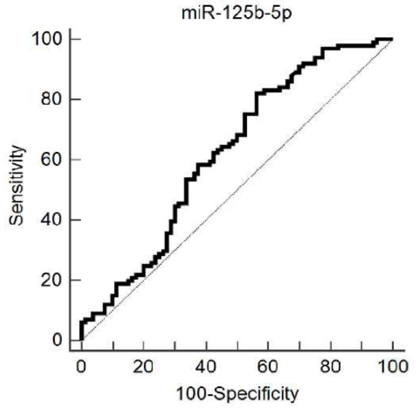

Area under the ROC curve (AUC)	0.625
Standard Error	0.0429
95% Confidence interval	0.550 to 0.695
Significance level P (Area=0.5)	0.0036

Estimated sensitivity at fixed specificity			
Specificity	Sensitivity	95% CI	Criterion
90.00	14.85	5.94 to 25.74	>0.007436

Criterion	Sensitivity	95% CI	Specificity	95% CI	+LR	95% CI	-LR	95% CI
>0.002223	82.18	73.3 - 89.1	43.75	32.7 - 55.3	1.46	1.2 - 1.8	0.41	0.3 - 0.7

G

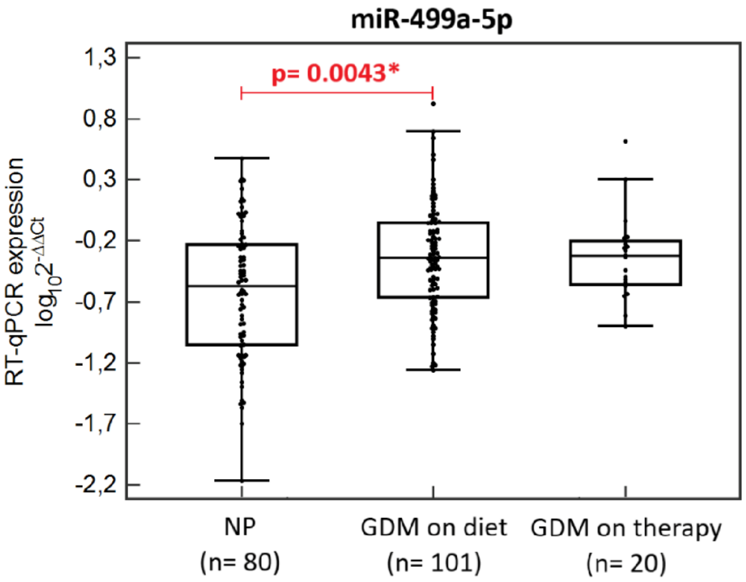

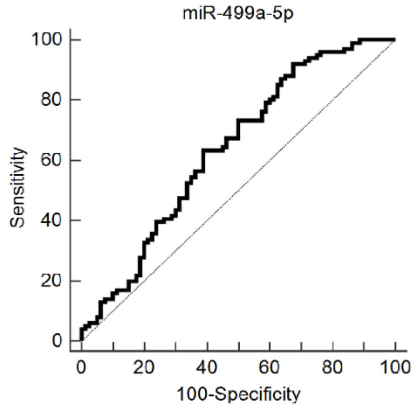

Area under the ROC curve (AUC)	0.637
Standard Error	0.0423
95% Confidence interval	0.562 to 0.707
Significance level P (Area=0.5)	0.0012

Estimated sensitivity at fixed specificity			
Specificity	Sensitivity	95% CI	Criterion
90.00	15.84	6.93 to 28.71	>1.191424

Criterion	Sensitivity	95% CI	Specificity	95% CI	+LR	95% CI	-LR	95% CI
>0.355789	63.37	53.2 - 72.7	61.25	49.7 - 71.9	1.64	1.2 - 2.2	0.60	0.4 - 0.8

H

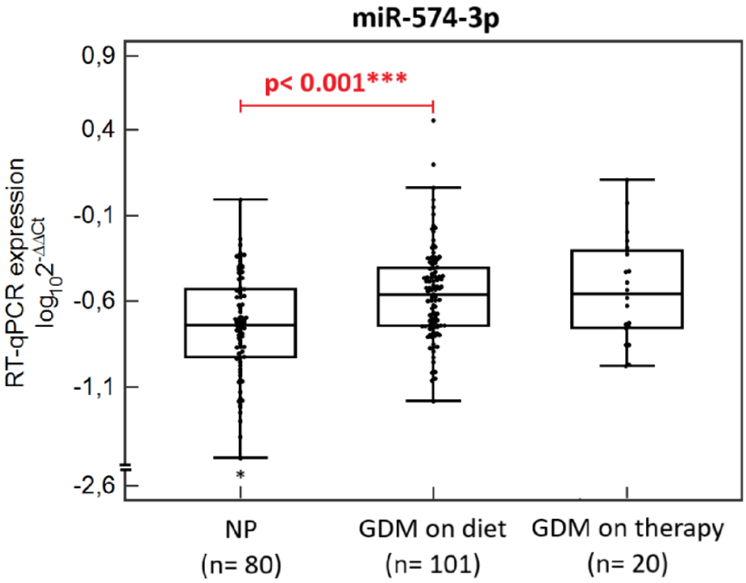

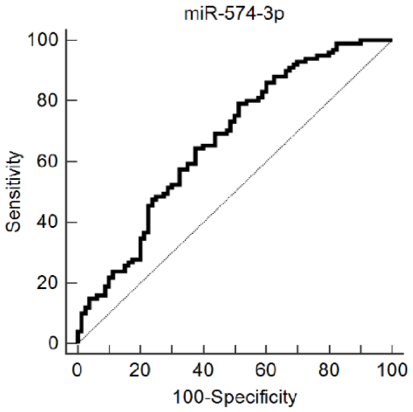

Area under the ROC curve (AUC)	0.672
Standard Error	0.0407
95% Confidence interval	0.598 to 0.740
Significance level P (Area=0.5)	<0.0001

Estimated sensitivity at fixed specificity			
Specificity	Sensitivity	95% CI	Criterion
90.00	21.78	11.88 to 35.54	>0.42623

Criterion	Sensitivity	95% CI	Specificity	95% CI	+LR	95% CI	-LR	95% CI
>0.175418	79.21	70.0 - 86.6	48.75	37.4 - 60.2	1.55	1.2 - 2.0	0.43	0.3 - 0.7

Supplementary Figure S2. Comparison of miRNAs gene expression in peripheral blood leukocytes in early stages of gestation between pregnancies with normal course and those with GDM with respect to the GDM treatment strategies (GDM pregnancies on diet only and GDM pregnancies requiring the combination of diet and administration of appropriate therapy). **A-C**, significant upregulation (after the Benjamini-Hochberg correction) of miR-20a-5p, miR-20b-5p, and miR-195-5p in both groups of pregnancies destinated to develop GDM irrespective of the treatment strategies (on diet only or on the combination of diet and therapy); **D-H**, significant upregulation (after the Benjamini-Hochberg correction) of miR-1-3p, miR-100-5p, miR-125b-5p, miR-499-5p, and miR-574-3p in the group of pregnancies destinated to develop GDM, which was well managed by diet only.
