# Supplementary material for: Cardiovascular Disease-Associated MicroRNAs as Novel Biomarkers of First-Trimester Screening for Gestational Diabetes Mellitus in the Absence of Other Pregnancy-Related Complications
Source: Int J Mol Sci. 2022 Sep 13;23(18):10635. doi: 10.3390/ijms231810635 (PMC9501303; doi:10.3390/ijms231810635)
Supplement: Supplementary file 1 [file ijms-23-10635-s001.zip › Supplementary Figure S3.pdf]

Supplementary Figure S3.

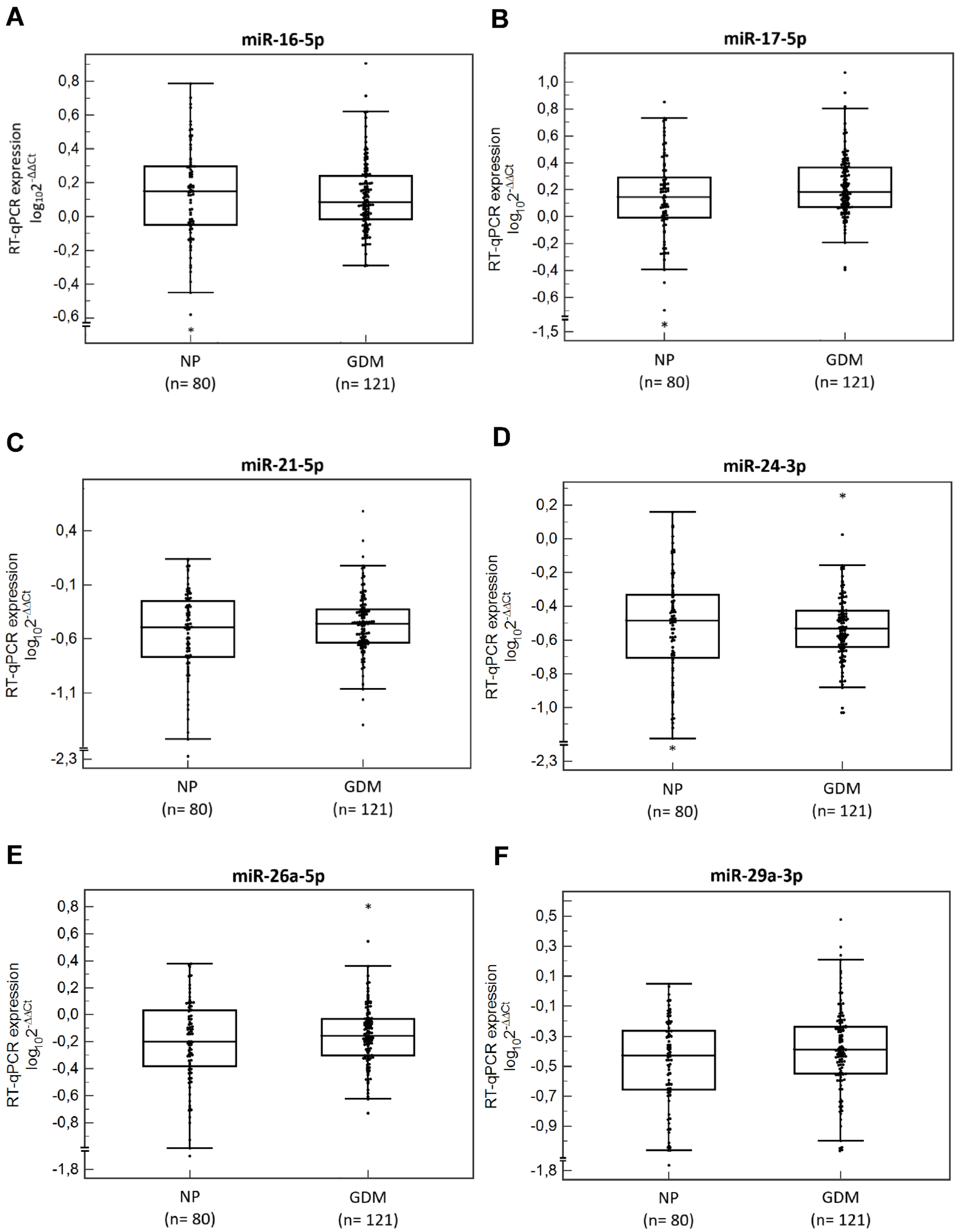

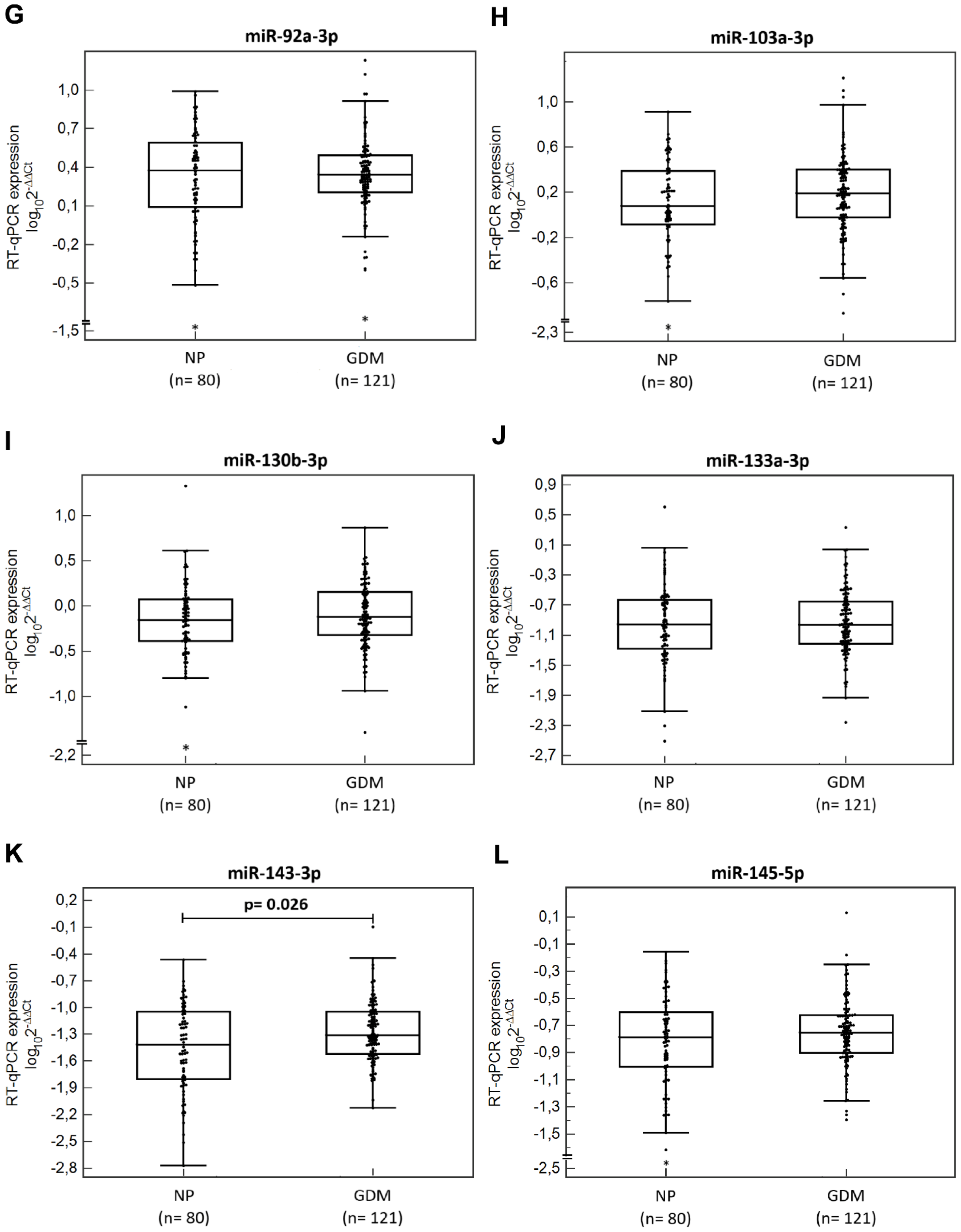
G
miR-92a-3p
RT-qPCR expression
$\log_{10}2^{-\Delta\Delta Ct}$
1,0
0,7
0,4
0,1
-0,2
-0,5
-1,5
NP
(n= 80)
GDM
(n= 121)
H
miR-103a-3p
1,0
0,6
0,2
-0,2
-0,6
-2,3
I
miR-130b-3p
1,0
0,5
0,0
-0,5
-1,0
-2,2
J
miR-133a-3p
0,9
0,5
0,1
-0,3
-0,7
-1,1
-1,5
-1,9
-2,3
-2,7
K
miR-143-3p
p= 0.026
0,2
-0,1
-0,4
-0,7
-1,0
-1,3
-1,6
-1,9
-2,2
-2,5
-2,8
L
miR-145-5p
0,1
-0,1
-0,3
-0,5
-0,7
-0,9
-1,1
-1,3
-1,5
-2,5

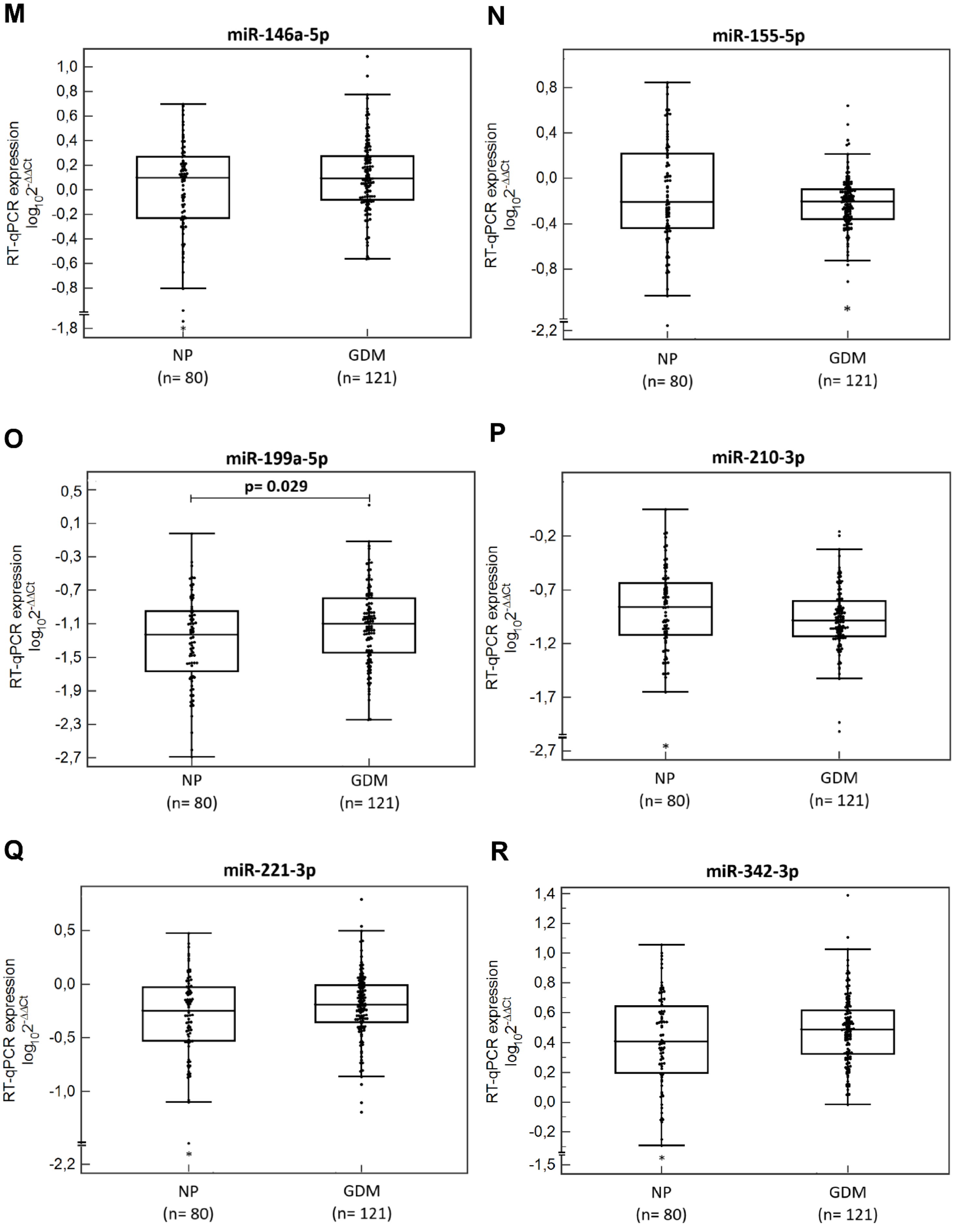

Supplementary Figure S3. Comparison of miRNAs gene expression in peripheral blood leukocytes in early stages of gestation between pregnancies with normal course and those with GDM – non-significant results after the Benjamini-Hochberg correction (but with $\alpha < 0.05$) and non-significant results ($\alpha > 0.05$).
