# Supplementary material for: Cardiovascular Disease-Associated MicroRNAs as Novel Biomarkers of First-Trimester Screening for Gestational Diabetes Mellitus in the Absence of Other Pregnancy-Related Complications
Source: Int J Mol Sci. 2022 Sep 13;23(18):10635. doi: 10.3390/ijms231810635 (PMC9501303; doi:10.3390/ijms231810635)
Supplement: Supplementary file 1 [file ijms-23-10635-s001.zip › Supplementary Figure S4.pdf]

Supplementary Figure S4.

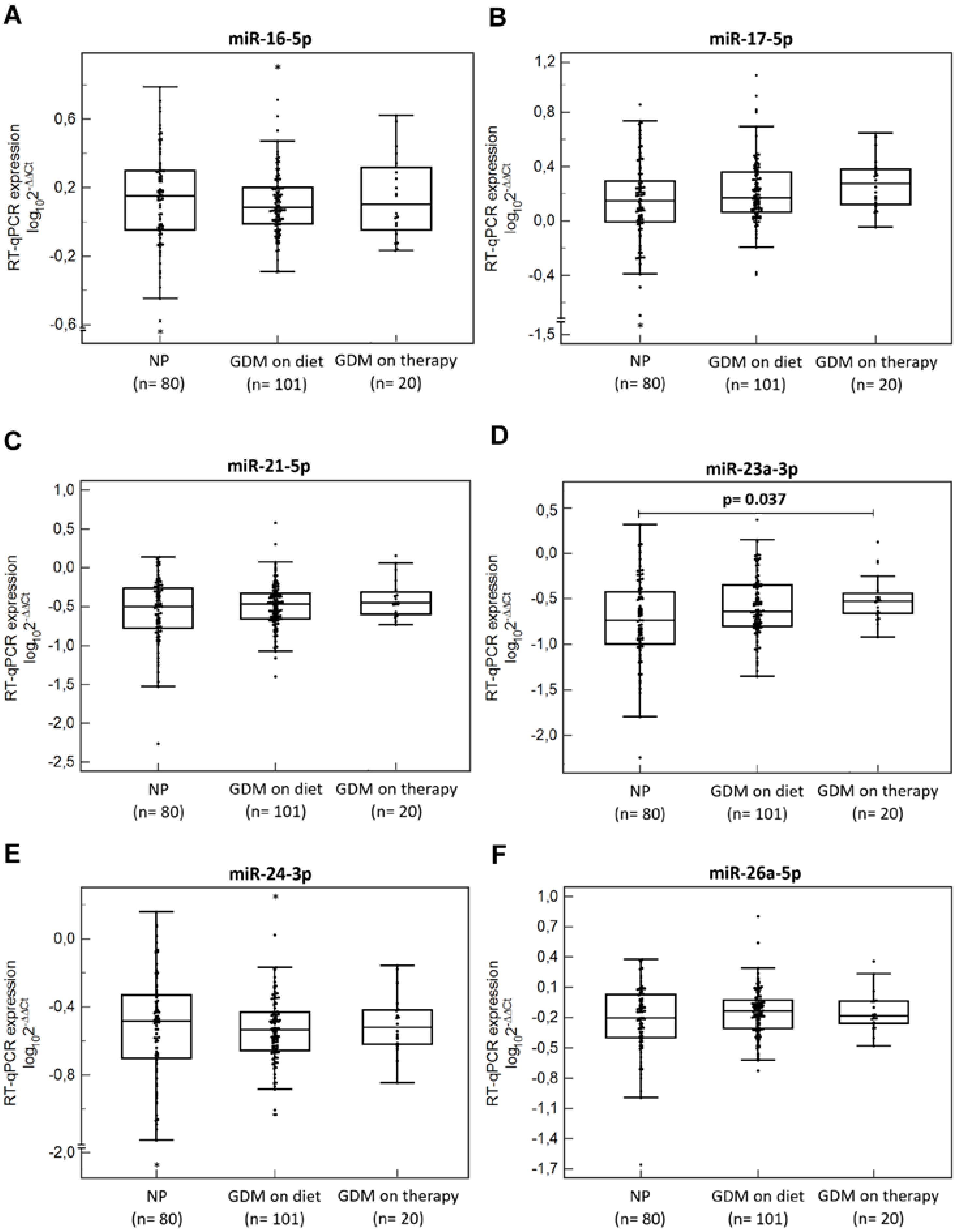

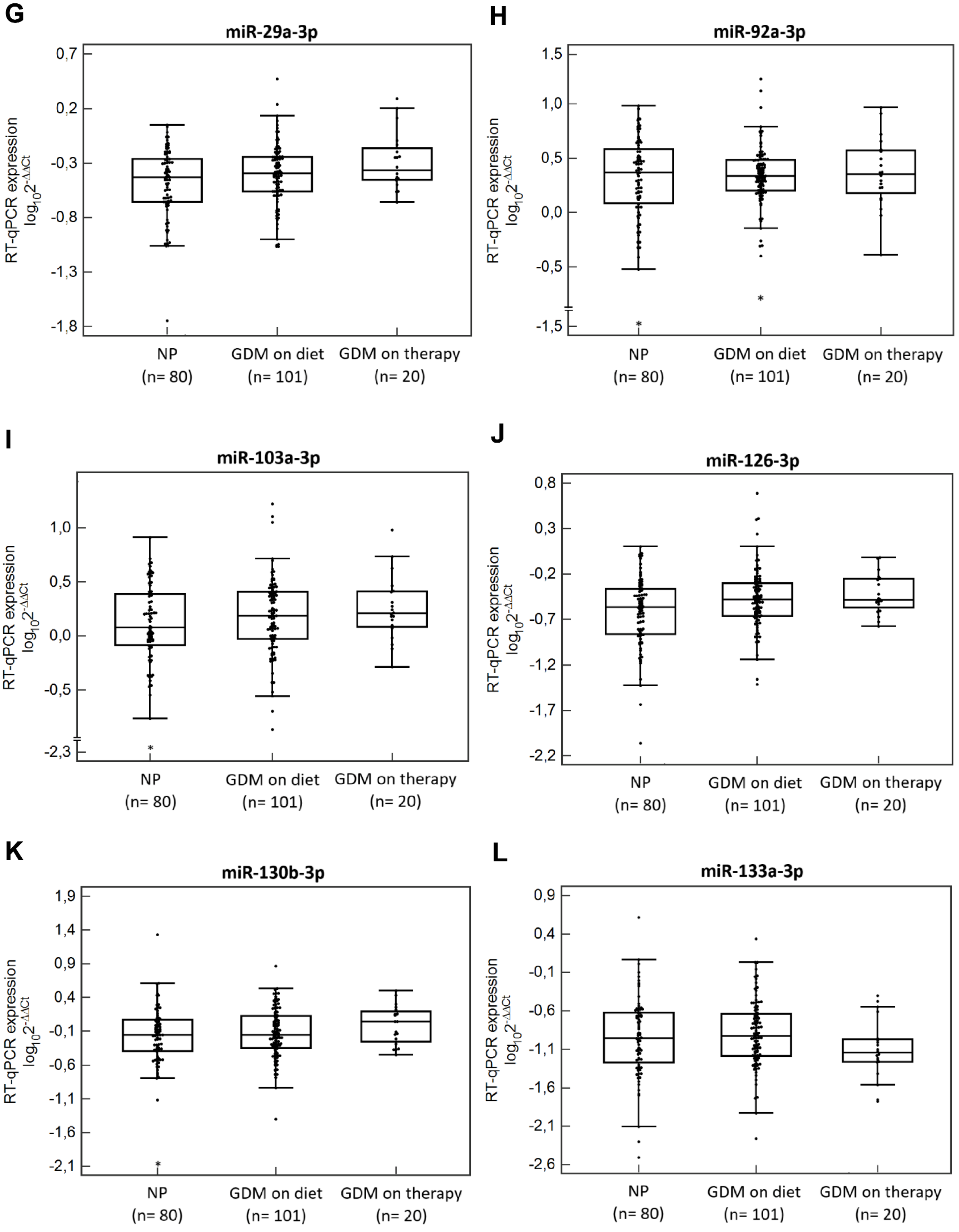
G
miR-29a-3p
RT-qPCR expression log10 2-ΔΔCt
0,7
0,2
-0,3
-0,8
-1,3
-1,8
NP (n= 80)
GDM on diet (n= 101)
GDM on therapy (n= 20)
H
miR-92a-3p
RT-qPCR expression log10 2-ΔΔCt
1,5
1,0
0,5
0,0
-0,5
-1,5
NP (n= 80)
GDM on diet (n= 101)
GDM on therapy (n= 20)
I
miR-103a-3p
RT-qPCR expression log10 2-ΔΔCt
1,0
0,5
0,0
-0,5
-2,3
NP (n= 80)
GDM on diet (n= 101)
GDM on therapy (n= 20)
J
miR-126-3p
RT-qPCR expression log10 2-ΔΔCt
0,8
0,3
-0,2
-0,7
-1,2
-1,7
-2,2
NP (n= 80)
GDM on diet (n= 101)
GDM on therapy (n= 20)
K
miR-130b-3p
RT-qPCR expression log10 2-ΔΔCt
1,9
1,4
0,9
0,4
-0,1
-0,6
-1,1
-1,6
-2,1
NP (n= 80)
GDM on diet (n= 101)
GDM on therapy (n= 20)
L
miR-133a-3p
RT-qPCR expression log10 2-ΔΔCt
0,9
0,4
-0,1
-0,6
-1,1
-1,6
-2,1
-2,6
NP (n= 80)
GDM on diet (n= 101)
GDM on therapy (n= 20)

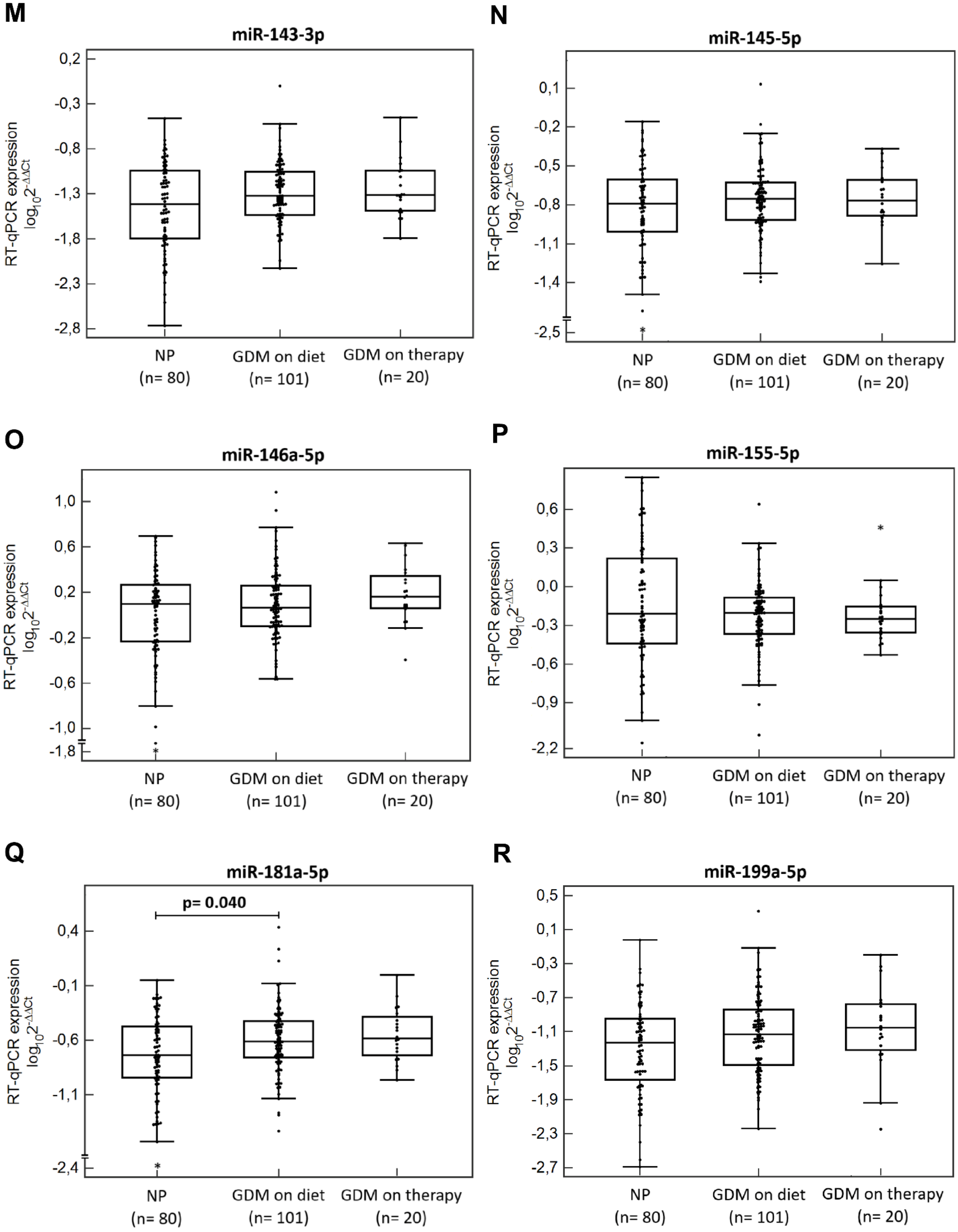
M
miR-143-3p
RT-qPCR expression
log10 2-ΔΔCt
0,2
-0,3
-0,8
-1,3
-1,8
-2,3
-2,8
NP (n= 80)
GDM on diet (n= 101)
GDM on therapy (n= 20)
N
miR-145-5p
RT-qPCR expression
log10 2-ΔΔCt
0,1
-0,2
-0,5
-0,8
-1,1
-1,4
-2,5
NP (n= 80)
GDM on diet (n= 101)
GDM on therapy (n= 20)
O
miR-146a-5p
RT-qPCR expression
log10 2-ΔΔCt
1,0
0,6
0,2
-0,2
-0,6
-1,0
-1,8
NP (n= 80)
GDM on diet (n= 101)
GDM on therapy (n= 20)
P
miR-155-5p
RT-qPCR expression
log10 2-ΔΔCt
0,6
0,3
0,0
-0,3
-0,6
-0,9
-2,2
NP (n= 80)
GDM on diet (n= 101)
GDM on therapy (n= 20)
Q
miR-181a-5p
p= 0.040
RT-qPCR expression
log10 2-ΔΔCt
0,4
-0,1
-0,6
-1,1
-2,4
NP (n= 80)
GDM on diet (n= 101)
GDM on therapy (n= 20)
R
miR-199a-5p
RT-qPCR expression
log10 2-ΔΔCt
0,5
0,1
-0,3
-0,7
-1,1
-1,5
-1,9
-2,3
-2,7
NP (n= 80)
GDM on diet (n= 101)
GDM on therapy (n= 20)

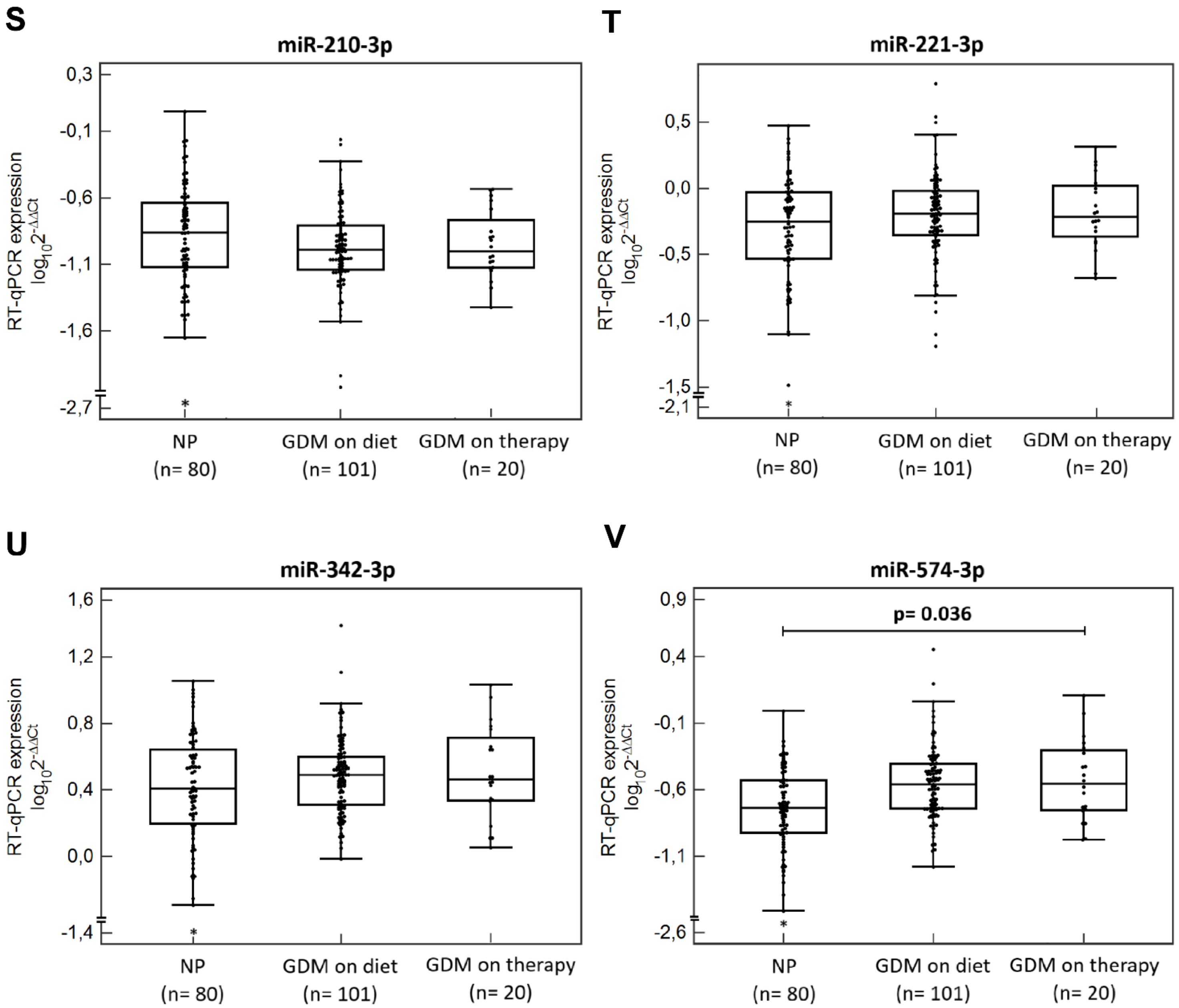

Supplementary Figure S4. Comparison of miRNAs gene expression in peripheral blood leukocytes in early stages of gestation between pregnancies with normal course and those with GDM with respect to the GDM treatment strategies (GDM pregnancies on diet only and GDM pregnancies requiring the combination of diet and administration of appropriate therapy) – non-significant results after the Benjamini-Hochberg correction (but with $\alpha < 0.05$) and non-significant results ($\alpha > 0.05$).
